# Gene expression analysis of potential genes and pathways involved in the pathogenic mechanisms of parvovirus B19 in human colorectal cancer

**DOI:** 10.3892/ol.2014.2151

**Published:** 2014-05-16

**Authors:** WEI-PING ZHANG, HUA YANG, HONG CHEN, HAI-RONG ZHU, QUAN LEI, YUN-HONG SONG, ZHONG-MING DAI, JING-SHAN SUN, LI-LI JIANG, ZHAN-GUO NIE

**Affiliations:** 1 Department of Gastroenterology and Hepatology, Urumqi Military General Hospital, Urumqi, Xinjiang 830000, P.R. China; 2 Department of Outpatients, Urumqi Military General Hospital, Urumqi, Xinjiang 830000, P.R. China; 3 Department of Blood Transfusion, Urumqi Military General Hospital, Urumqi, Xinjiang 830000, P.R. China; 4 Department of Medical Administration, Urumqi Military General Hospital, Urumqi, Xinjiang 830000, P.R. China

**Keywords:** colorectal cancer, parvovirus B19, pathogenesis, microarray, human

## Abstract

In order to investigate the pathogenic mechanisms of parvovirus B19 in human colorectal cancer, plasmids containing the VP1 or VP2 viral capsid proteins or the NS1 non-structural proteins of parvovirus B19 were constructed and transfected into primary human colorectal epithelial cells and LoVo cells. Differential gene expression was detected using a human genome expression array. Functional gene annotation analyses were performed using Database for Annotation, Visualization and Integrated Discovery v6.7 software. Gene ontology (GO) analyses revealed that VP1-related functions included the immune response, immune system process, defense response and the response to stimulus, while NS1-associated functions were found to include organelle fission, nuclear division, mitosis, the M-phase of the mitotic cell cycle, the mitotic cell cycle, M-phase, cell cycle phase, cell cycle process and cell division. Pathway expression analysis revealed that VP1-associated pathways included cell adhesion molecules, antigen processing and presentation, cytokines and the inflammatory response. Moreover, NS1-associated pathways included the cell cycle, pathways in cancer, colorectal cancer, the wnt signaling pathway and focal adhesion. Among the differential genes detected in the present study, 12 genes were found to participate in general cancer pathways and six genes were observed to participate in colorectal cancer pathways. NS1 is a key molecule in the pathogenic mechanism of parvovirus B19 in colorectal cancer. Several GO categories, pathways and genes were selected and may be the key targets through which parvovirus B19 participates in colorectal cancer pathogenesis.

## Introduction

Colorectal cancer is the third most common type of cancer and the second most frequent cause of cancer mortality in numerous industrialized countries (1). The majority of tumors arise sporadically with no clear cause or genetic predisposition. Several risk factors have been considered as causes of colorectal cancer, but little has been confirmed. Viruses are among the few known causes of cancer and contribute to various malignancies worldwide (2). Previous studies on viral etiology in colon cancer have reported contradictory findings (3,4).

Parvovirus B19 (B19) is a non-enveloped virus with a linear, single-stranded DNA genome. The B19 viral genome encodes three proteins: The non-structural protein, NS1, and two viral capsid proteins, VP1 and VP2 (5). In our previous study, significantly higher levels of B19 nucleic acids and proteins were found in neoplastic colon tissues (6). This finding indicates that an association may exist between B19 infection and the development of colon neoplasia.

Infection with parvovirus B19 is a global concern. The infection rate is similar in the United States, Europe and Asia, with ~50% of 15-year-old adolescents and >60% of adults being seropositive (5). A previous study has shown that B19 infection may contribute to the pathogenesis of acute lymphoblastic and myeloblastic leukemia (7). However, few studies have investigated B19 in solid tumors or the mechanisms or regulatory proteins that could be involved. Therefore, it is important to establish whether B19 contributes to the pathogenesis of colorectal cancer and its underlying mechanism.

The present study aimed to investigate the pathogenic mechanisms underlying B19 in colon carcinoma by analyzing differential gene expression and biological functions, through assessing the changes in primary human colorectal epithelial cells (HCECs) and LoVo cells following transfection with plasmids containing VP1, VP2 and NS1.

## Materials and methods

### Plasmid construction

The recombinant eukaryotic cell inducible expression vectors, pReceiver-M03-VP1, pReceiver-M33-VP2 and pReceiver-M16-NS1, were constructed by inserting B19 full-length VP1, VP2 and NS1 complementary DNA into pReceiver-M03, pReceiver-M33 or pReceiver-M16 (GeneCopoeia, Inc., Rockville, MD, USA), respectively. The VP1, VP2 and NS1 sequences were amplified by polymerase chain reaction (PCR) analysis using the pGEM/1-B19 plasmid provided by Professor J.P. Clewley at the Central Public Health Laboratory (London, UK) as the template, which contained the B19 full-length open reading frame. The presence of the recombinant plasmid was confirmed using DNA sequencing.

### Cell culture and transfection

Samples of normal human colon tissue >10 cm distant to the tumors was obtained from patients with colorectal adenocarcinoma. HCECs were isolated from the normal human colorectal tissue and were washed several times using phosphate-buffered saline (PBS) containing penicillin, streptomycin and amphotericin B using thermolysin and collagenase type I (Sigma-Aldrich, St. Louis, MO, USA), as described previously (8). The cells were then cultured in Epithelial Cell Growth Medium-2 (ScienCell Research Laboratories, Carlsbad, CA, UDA) containing amphotericin B. LoVo carcinoma cells were obtained from the American Type Culture Collection (Rockville, MD, USA) and were cultured in Dulbecco’s modified Eagle’s medium containing 10% fetal bovine serum. All cells were cultured at 37°C in an atmosphere containing 5% CO_2_. All procedures were performed in accordance with standard guidelines for the study of humans and were approved by the Research Ethics Committee of Urumqi Military General Hospital (Urumqi, China). All patients provided written informed consent.

The cells were transfected with pReceiver-M03-VP1, pReceiver-M33-VP2 and pReceiver-M16-NS1, using pReceiver-M03, pReceiver-M33 and pReceiver-M16 as controls. Transfection was performed using Lipofectamine^®^ LTX and PLUS™ Reagents (Invitrogen Life Technologies, Carlsbad, CA, USA) according to the manufacturer’s instructions, with untransfected cells used as blank controls.

### Fluorescence microscopy

The expression of enhanced green fluorescent protein (eGFP), enhanced cyan fluorescent protein (eCFP) and enhanced yellow fluorescent protein (eYFP) in the transfected HCECs and LoVo cells was observed using a fluorescence microscope (TE2000-U, Nikon Corporation, Tokyo, Japan) equipped with a fluorescence filter. Digital images of the cells were captured using a spot camera system (Nikon Corporation).

### Flow cytometric analysis

The cells (1×10^6^) were fixed in 75% alcohol for 12–16 h at 4°C, followed by ethidium bromide (50 μg/ml) and RNase (100 μg/ml) treatment at 25°C for 30 min. Analysis was performed using a flow cytometer (FACScan; Becton Dickinson, Bedford, MA, USA).

### Reverse transcription PCR (RT-PCR) analysis

RNA was extracted 24 h and 48 h subsequent to transfection, and RT-PCR was performed. The following primer pairs were used: Vp1 forward, ttctgcatgactgctactgga and reverse, atc ccctagaaaacccatcct; Vp2 forward, tatttgaggaggtggctgatg and reverse, ccaataaaggaacccagcaat; Ns1 forward, ggtggtctggga tgaaggtat and reverse, gtgttcccgcttacaacaaaa; and glyceraldehyde-3-phosphate dehydrogenase (GAPDH). forward, tcggagtcaacggatttggtcgta and reverse, tggcatggactgtggtcatgagtc.

### Western blot analysis

Protein extraction was performed by washing the cells twice with ice-cold PBS, followed by homogenization in lysis buffer [50 mM HEPES (pH 7.5), 150 mM NaCl, 10% glycerol, 1% Triton X-100, 1.5 mM MgCl_2_, 1 mM EDTA, 10 mM Na_4_(PO_4_)_2_, 25 *μ*g/ml aprotinin and 25 *μ*g/ml leupeptin] at 24 h, 48 h and 72 h post-transfection. The insoluble fraction was removed using centrifugation at 1,000 × g for 15 min at 4°C. Proteins were analyzed using electrophoresis (50 μg per lane) on 10% polyacrylamide gels and transferred to polyvinylidene fluoride (PVDF) membranes. Mouse monoclonal antibodies against the B19 proteins VP1 and VP2 (clone, R92F6; Chemicon, Billerica, MA, USA) and anti-NS1 antibodies [a gift from Professor Susanne Modrow (9) and Dr Simon Bredl, Institute of Medical Microbiology, University of Regensburg, Germany] were used to identify the proteins on the PVDF membranes. Horseradish peroxidase-conjugated goat antimouse secondary antibodies (Sigma-Aldrich) were detected using enhanced chemiluminescence western blot analysis reagents (Pierce Biotechnology, Inc., Rockford, IL, USA).

### Microarray hybridization and data analysis

Microarray hybridization was performed by Shanghai Biochip Co., Ltd., (Shanghai, China) using an Agilent SurePrint G3 Human GE 8×60k microarray (Agilent Technologies, Santa Clara, CA, USA) that targeted 27,958 Entrez Gene RNAs and 7,419 long non-coding RNAs (reference). In brief, total RNAs from the transfected cells were extracted and purified using the Qiagen RNeasy^®^ Mini kit (Qiagen, Hilden, Germany). Total RNA was amplified using the Low Input Quick Amp Labeling kit, One-Color (Agilent Technologies). For hybridization, each slide was hybridized with 1.65 μg Cy-3 labeled complementary RNA using the Gene Expression Hybridization kit (Agilent Technologies) in a Hybridization Oven (Agilent Technologies) according to the manufacturer’s instructions. Subsequent to 17 h of hybridization, the slides were washed in staining dishes (Thermo Fisher Scientific, Waltham, MA, USA) with Gene Expression Wash Buffer (Agilent Technologies) according to the manufacturer’s instructions. The slides were scanned at a 3-μm resolution using the green dye channel in an Agilent Microarray Scanner (Agilent Technologies). The data were read using Feature Extraction Software 10.7 (Agilent Technologies), and were normalized using Quantile Algorithm, Gene Spring 11.0 software (Agilent Technologies).

The data from three replicates were averaged. Genes were defined as differentially expressed if the intensity ratio (Cy5) was found to increase or decrease >2-fold and if the intensity ratio (Cy5) showed the same direction of change (upregulated or downregulated) in all three experimental repeats. Gene ontology (GO) and pathway analyses were performed using Database for Annotation, Visualization and Integrated Discovery v6.7 software (10,11).

### Quantitative (q)PCR analysis

qPCR analysis using SYBR^®^ Green (Invitrogen Life Technologies) was performed in order to verify the results of the microarray analysis. Total RNA was extracted from the transfected cells. The RNA was reverse transcribed using Murine Leukemia Virus reverse transcriptase (Promega Corp., Madison, WI, USA). The expression of the 12 genes that were identified as being associated with apoptosis in the microarray analysis was determined using qPCR analysis with SYBR-Green I (Invitrogen Life Technologies). GAPDH was used as an internal control and distilled water was used as a negative control. The amplification reaction consisted of 10X PCR buffer, 1.25 units of JumpStart™ Taq (Sigma-Aldrich), 10 pmol forward and reverse primers, 0.2 μmol dNTP, 100 ng template and 0.2X SYBR-Green I (Amresco Inc., Solon, OH, USA) in a final volume of 50 μl. The reactions were performed using the StepOne^TM^ Real-Time PCR System (Applied Biosystems, Inc., Foster City, CA, USA). The mRNA expression of the 12 genes was normalized with GAPDH using the 2^−ΔΔCt^ method (12). The primer sequences used for GAPDH and the 12 genes were retrieved from PrimerBank (http://pga.mgh.harvard.edu/primerbank/).

## Results

### VP1, VP2 and NS1 expression in HCECs and LoVo cells

**Figure 1:**
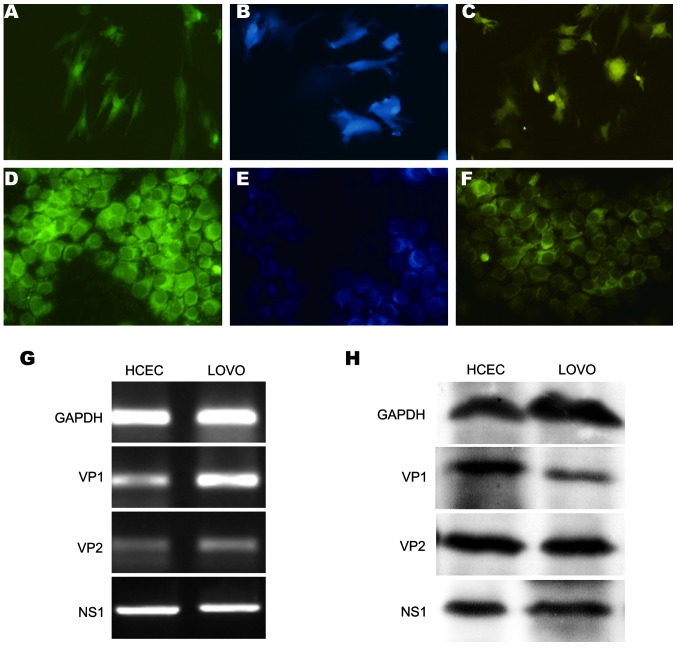
Expression of VP1, VP2 and NS1 in HCEC and LoVo cells. (A–C) HCECs and (D–F) LoVo cells marked with green, cyan and yellow fluorescence (magnification, ×400). (G) Reverse transcription polymerase chain reaction (RT-PCR) analysis revealing VP1, VP2 and NS1 mRNA expression in the HCECs and LoVo cells. (H) Western blot analysis showing VP1, VP2 and NS1 protein expression in the HCECs and LoVo cells. HCECs, human colorectal epithelial cells.

Primary normal HCECs were isolated, cultured and transiently transfected with the pReceiver-M03-VP1, pReceiver-M33-VP2 and pReceiver-M16-NS1 constructs. The expression of eGFP-VP1, eCFP-VP2 and eYFP-NS1 in the HCECs and LoVo cells was analyzed and confirmed using fluorescence microscopy, RT-PCR analysis and western blot analysis. Marked green, cyan and yellow fluorescence, indicating the expression of VP1, VP2 and NS1, respectively, was observed at 24 h post-transfection, with the strongest expression observed after 48 h (Fig. 1A–F). The mRNA expression of VP1, VP2 and NS1 was detected using RT-PCR analysis in the transfected cells (Fig. 1G). To further assess the expression of eGFP-VP1, eCFP-VP2 and eYFP-NS1 in the HCECs and LoVo cells, the protein expression of VP1, VP2 and NS1 was confirmed using anti-VP1, -VP2 and -NS1 antibodies in western blot analysis (Fig. 1H).

### Flow cytometric analysis

No significant changes in the cell cycle or apoptosis were identified in the HCECs transfected with pReceiver-M03-VP1, pReceiver-M33-VP2 or pReceiver-M16-NS1 and pReceiver-M03, pReceiver-M33 or pReceiver-M16. Similarly, no significant changes in cell cycle or apoptosis were identified in the LoVo cells transfected with pReceiver-M03-VP1, pReceiver-M33-VP2 or pReceiver-M16-NS1 and pReceiver-M03, pReceiver-M33 or pReceiver-M16.

### Differential gene analysis

Using the human genome expression microarray, differential gene expression was detected in the HCECs and LoVo cells transfected with pReceiver-M03-VP1, pReceiver-M33-VP2 or pReceiver-M16-NS1 compared with those transfected with pReceiver-M03, pReceiver-M33 or pReceiver-M16, respectively. The number of upregulated and downregulated genes (P<0.05; false discovery rate <0.05; fold-change >2.0) are shown in Table I. The top five differential genes in the six groups are shown in Table II. The fold change of the differentially-expressed genes associated with colorectal cancer are shown in Table III.

### GO analysis

The differentially-expressed genes were classified into different functional categories based on GO analysis for biological process, molecular function and cellular components. The primary GO categories for the upregulated genes in the HCECs and LoVo cells transfected with pReceiver-M03-VP1 included immune response, immune system process, defense response and response to stimulus, and for the downregulated genes was primarily cellular amino acid metabolic process. The predominant GO categories for the upregulated genes in the HCECs and LoVo cells transfected with pReceiver-M16-NS1 included organelle fission, nuclear division, mitosis, the M-phase of the mitotic cell cycle, the mitotic cell cycle, M-phase, cell cycle phase, cell cycle process and cell division (Table IV).

### Pathway analysis

Significant pathways for the upregulated and downregulated differentially-expressed genes are shown in Table V. No pathways or specified pathways were found among the upregulated genes in the HCECs following transfection with pReceiver-M33-VP2 or in the LoVo cells following transfection with pReceiver-M33-VP2 or pReceiver-M16-NS1 (Table V). Similarly, no pathways were found among the downregulated genes in the HCECs following transfection with pReceiver-M03-VP1, pReceiver-M33-VP2 or pReceiver-M16-NS1, or in the LoVo cells following transfection with pReceiver-M03-VP1 or pReceiver-M33-VP2 compared with the cells transfected with the control plasmids.

### Confirmation of microarray results using qPCR analysis

To verify the microarray analysis data, the expression of the 12 differentially-expressed genes selected using microarray analysis was confirmed by qPCR analysis in the different groups. Consistent results were observed with regard to the nine genes in the microarray and qPCR analysis data (Table VI).

## Discussion

Despite our current understanding of the genetic alterations associated with the progression of colon cancer, the specific etiology of colorectal cancer has yet to be elucidated. Epidemiological studies have indicated that environmental factors and host immunological characteristics may contribute to the initiation and progression of colon cancer. Infectious agents, primarily viral infection, are acquired through the environment and have the potential to alter numerous regulatory processes, which may result in the development of colorectal cancer. Our previous study showed that B19 infection may cause colon carcinoma (6). However, little is known regarding the pathogenic mechanisms responsible for B19-induced tumorigenesis.

B19 was discovered in 1974 and is the only Parvoviridae family member that is known to be pathogenic in humans. The genome of B19 has two large open reading frames encoding a single non-structural protein, NS1, and two capsid proteins, VP1 and VP2, which form an icosahedral capsid (5). The contribution of these viral proteins to B19 infectivity have yet to be experimentally demonstrated due to problems with *in vitro* culture and the lack of an infectious clone. Due to the difficulty in culturing B19 *in vitro*, little experimental evidence exists regarding the known and putative roles of B19 viral proteins in infectivity. In the present study, plasmids containing VP1, VP2 and NS1 were constructed and transfected into cultured HCECs and LoVo cells. Through the analysis of differentially-expressed genes and their functional enrichment, the present study aimed to identify potential targets to enable further investigation of the function of B19 in colon carcinoma, rather than to identify specific signaling pathways or molecules leading to colon carcinoma in which B19 participated.

Current understanding of the B19 viral proteins is primarily based on studies of other parvoviruses. The B19 NS protein is a multifunctional protein, for which sequence analysis has revealed that, in addition to transregulation of the p6 promoter (13,14), NS contains motifs for nucleoside triphosphate (NTP) binding and hydrolysis (15) associated with helicase activity, thus indicating a role for the protein in B19 DNA replication. A previous study has also indicated that the NTP-binding motifs of NS play roles in the induction erythroid lineage cell apoptosis during B19 infection (16). VP2 is the major capsid protein, comprising 95% of the capsid and 58-kDa in size (17). Previous studies in insect cells have reported that VP2 can self-assemble into virus-like particles (17) and that it is capable of binding directly to blood group P antigen, which is the cellular receptor of B19 (18). VP1 is the minor capsid protein, which has an identical amino acid sequence to VP2, plus an extra 227 amino acids termed the VP1-unique region (VP1u)) at the amino terminus (19). Previous studies have shown that the VP1u, which is found on the exterior of the capsid, contains the primary neutralizing epitopes of B19 (20–22). Furthermore, a conserved phospholipase A2 motif has been identified in the VP1u of members of the Parvoviridae family, including B19 (23,24). Two small 7.5- and 11-kDa proteins, are encoded by the small abundant mRNA of B19 (25–27) and are unique among those parvoviruses that have so far been characterized. A number of proline-rich motifs are contained within the 11-kDa protein and are conserved to the Src homology 3 binding domain of eukaryotic proteins (28); however, the function of the 7.5- and 11-kDa proteins in B19 replication and/or pathogenesis has yet to be elucidated.

In the present study, plasmids containing VP1, VP2 and NS1 were constructed for transfection into cultured HCECs and LoVo cells. Hundreds of differentially-expressed genes were identified in the HCECs and LoVo cells following VP1, VP2 and NS1 protein expression in different ontological pathways and functional GO groups. GO analyses revealed that the significant VP1-related ontology categories included that of immune response, immune system process, defense response and response to stimulus, while significant NS1-related ontology categories included organelle fission, nuclear division, mitosis, M-phase of the mitotic cell cycle, mitotic cell cycle, M-phase, cell cycle phase, cell cycle process and cell division. Pathway expression analysis identified that VP1-related pathways included cell adhesion molecules, antigen processing and presentation, cytokines and inflammatory response. Pathway expression analysis identified that NS1-related pathways included cell cycle, pathways in cancer, colorectal cancer, the wnt signaling pathway and focal adhesion. The functional GO categories and pathways associated with VP1 and NS1 that were identified in the present study were consistent with the functions of VP1 and NS1 reported previously (6,9,13,14,16–23,28–30). This indicates that NS1 has a significant role in the pathogenesis of B19 in colorectal carcinoma.

In conclusion, the present study identified twelve differentially-expressed genes (BAX, EP300, BMP4, FZD1, IGF1, PPARG, PIAS4, RAC3, RUNX1T1, TCEB2, TCF7L2 and FOS) that were found to participate in general cancer pathways, and six genes (BAX, APC, RAC3, FZD1, TCF7L2 and FOS) that were found to specifically participate in colorectal cancer pathways. Furthermore, genes associated with cancer, including MYCL1, APCDD1, VTI1A, TP53INP2, TP53I11, TP53INP1, CRCX7, TMBIM1, LRP11, CCND1, FOSB, FOSL1, FZD4 and FZD10, were found to be differentially expressed. These may be the primary genes involved in regulating the pathogenesis of B19 in colorectal carcinoma. Moreover, NS1 may be the key molecule involved in the pathogenesis of B19 in colorectal carcinoma.

## Figures and Tables

**Table I:** Number of differentially-expressed genes in HCECs and LoVo cells transfected with plasmids.

	VP1		VP2		NS1	
			
Cell	Upregulated, n	Downregulated, n	Upregulated, n	Downregulated, n	Upregulated, n	Downregulated, n
HCEC	740	361	131	124	447	690
LoVo	613	328	546	562	508	652

HCECs, human colorectal epithelial cells.

**Table II:** Top five differentially-expressed genes in HCECs and LoVo cells.

	Upregulated				Downregulated			
		
Group	Gene symbol	Gene name	Gene ID	Log^2^ ratio	Gene symbol	Gene name	Gene ID	Log^2^ ratio
HCEC								
VP1								
	NME4	Non-metastatic cells 4, protein expressed in	NM_005009	10.9	RWDD4A	RWD domain containing 4A	NM_152682	−7.7
	TMCO1	Transmembrane and coiled-coil domains 1	NM_019026	5.9	POU3F3	POU class 3 homeobox 3	NM_006236	−4.0
	IL6	Interleukin 6	NM_000600	5.1	ABCC6P1	ATP-binding cassette, sub-family C, member 6 pseudogene 1	NR_003569	−3.8
	CCL20	Chemokine (C-C motif) ligand 20	NM_004591	5.1	LOC100131599	Hypothetical protein LOC100131599	AK126221	−3.6
	CCL5	Chemokine (C-C motif) ligand 5	NM_002985	5.1	MYBPC2	Myosin-binding protein C, fast type	NM_004533	−3.2
VP2								
	TSPAN32	Tetraspanin 32	NM_139022	4.9	NME4	Non-metastatic cells 4, protein expressed in	NM_005009	−11.2
	LOC100294501	Hypothetical protein LOC100294501	XM_002344054	4.1	TMCO1	Transmembrane and coiled-coil domains 1	NM_019026	−6.1
	BTBD19	BTB (POZ) domain containing 19	NM_001136537	3.7	BTN1A1	Butyrophilin, subfamily 1, member A1	NM_001732	−5.0
	TTC29	Tetratricopeptide repeat domain 29	NM_031956	3.7	LOC221122	Hypothetical LOC221122	NR_026681	−4.5
	CCDC114	Coiled-coil domain containing 114	NM_144577	3.6	HIBCH	3-hydroxyisobutyryl-Coenzyme A hydrolase	NM_014362	−4.2
NS1								
	ATG9A	ATG9 autophagy related 9 homolog A (S. cerevisiae)	NM_001077198	11.4	SAE1	SUMO1-activating enzyme subunit 1	NM_005500	−6.1
	MTUS1	Microtubule associated tumor suppressor 1	NM_001001925	5.9	RFESD	Rieske (Fe-S) domain containing	NM_173362	−5.9
	UBE2CBP	Ubiquitin-conjugating enzyme E2C binding protein	NM_198920	5.9	GLYATL2	Glycine-N-acyltransferase-like 2	NM_145016	−5.7
	DNAJC8	DnaJ (Hsp40) homolog, subfamily C, member 8	NM_014280	5.4	C6ORF225	Chromosome 6 open reading frame 225	NM_001033564	−5.6
	C7ORF62	Chromosome 7 open reading frame 62	NM_152706	5.2	MRPS18A	Mitochondrial ribosomal protein S18A	NM_018135	−5.4
LoVo								
VP1								
	IFI6	Interferon, α-inducible protein 6	NM_022873	4.8	WDD4A	RWD domain containing 4A	NM_152682	−7.3
	CCL5	Chemokine (C-C motif) ligand 5	NM_002985	4.7	HIBCH	3-hydroxyisobutyryl-Coenzyme A hydrolase	NM_014362	−4.4
	IL6	Interleukin 6 (interferon, β2)	NM_000600	4.7	POU3F3	POU class 3 homeobox 3	NM_006236	−4.1
	CCL20	Chemokine (C-C motif) ligand 20	NM_004591	4.7	ABCC6P1	ATP-binding cassette, sub-family C, member 6 pseudogene 1	NR_003569	−3.8
	TSPAN32	Tetraspanin 32	NM_139022	4.6	LOC100131599	Hypothetical protein LOC100131599	AK126221	−3.6
VP2								
	LOC283482	Hypothetical LOC283482	AK092513	6.3	KRTAP21-1	Keratin-associated protein 21-1	NM_181619	−6.8
	RRP15	Ribosomal RNA processing 15 homolog (*S. cerevisiae*)	NM_016052	6.2	MDM1	Mdm1 nuclear protein homolog (mouse)	NM_020128	−6.1
	TRIM71	Tripartite motif-containing 71	NM_001039111	5.9	C2ORF76	Chromosome 2 open reading frame 76	NM_001017927	−5.8
	ADPRHL1	ADP-ribosylhydrolase like 1	NM_138430	5.7	OR4K17	Olfactory receptor, family 4, subfamily K, member 17	NM_001004715	−5.8
	GOLGA6L6	Golgi autoantigen, golgin subfamily a, 6-like 6	NM_001145004	5.3	PAQR5	Progestin and adipoQ receptor family member V	NM_001104554	−5.6
NS1								
	TMEM50A	Transmembrane protein 50A	NM_014313	11.7	SAE1	SUMO1 activating enzyme subunit 1	NM_005500	−6.1
	TBCA	Tubulin folding cofactor A	NM_004607	9.0	RFESD	Rieske (Fe-S) domain containing	NM_173362	−5.9
	LOC723809	Hypothetical LOC723809	NR_027374	8.8	LOC100129954	Hypothetical LOC100129954	XM_001714109	−5.9
	PDZD8	PDZ domain containing 8	NM_173791	6.8	C9ORF38	Chromosome 9 open reading frame 38	AF090921	−5.4
	FEM1C	Fem-1 homolog c (*C. elegans*)	NM_020177	6.3	GORAB	Golgin, RAB6-interacting	NM_152281	−5.4

HCEC, human colorectal epithelial cell.

**Table III:** Fold-change of differentially-expressed genes associated with colorectal cancer.

		HCECs			LoVo cells		
			
Gene symbol	Genbank accession	VP1	VP2	NS1	VP1	VP2	NS1
RAC3	NM_005052	-	-	1.44	-	-	1.59
MYCL1	NM_005376	-	-	1.29	-	-	1.06
APCDD1	NM_153000	-	2.94	-	2.91	-	-
APC	NM_001127511	-	-	1.41	-	-	−2.38
TCF7L2	NM_030756	-	-	-	-	1.38	3.43
VTI1A	NM_145206	-	-	−1.17	-	-	−1.23
TP53INP2	NM_021202	1.47	-	-	-	-	-
TP53I11	NM_001076787	-	-	−3.42	-	-	−1.34
TP53INP1	NM_033285	-	-	-	1.08	-	-
CRCX7	NM_020311	1.77	-	-	1.63	-	-
BAX	NM_004324	-	-	1.23	-	-	1.05
TMBIM1	NM_022152	-	-	-	1.61	-	-
LRP11	NM_032832	-	-	-	-	3.04	-
VEGFA	NM_001025370	-	-	1.19	-	-	-
CCND1	NM_053056	-	-	2.59	-	-	-
FOS	NM_005252	-	-	−1.82	-	-	−1.83
FOSB	NM_006732	1.66	-	-	1.27	-	-
FOSL1	NM_005438	2.21	-	-	1.98	-	2.27
ID1	NM_002165	1.24	-	-	-	-	-
FZD4	NM_012193	3.40	-	-	2.85	-	-
FZD1	NM_003505	-	-	−1.14	-	-	−1.11
FZD10	NM_007197	1.15	-	-	-	-	-

-, no significant difference (log^2^ratio >1 or <−1); HCECs, human colorectal epithelial cells.

**Table IV:** GO terms for the differentially-expressed genes.

Group	GO term	Genes, n	Genes, %	Fold enrichment	P-value	FDR
Upregulation						
VP1 in HCEC	GO:0006955~immune response	87	11.85	3.48	9.34E-25	0.000
	GO:0002376~immune system process	105	14.31	2.90	1.06E-23	0.000
	GO:0006952~defense response	64	8.72	2.87	5.31E-14	0.000
	GO:0050896~response to stimulus	197	26.84	1.55	3.60E-12	0.000
VP2 in HCEC	No significant enrichment	-	-	-	-	-
NS1 in HCEC	GO:0048285~organelle fission	20	4.49	4.33	1.90E-07	0.000
	GO:0000280~nuclear division	19	4.27	4.28	4.96E-07	0.000
	GO:0007067~mitosis	19	4.27	4.28	4.96E-07	0.000
	GO:0000087~M-phase of mitotic cell cycle	19	4.27	4.20	6.43E-07	0.001
	GO:0000278~mitotic cell cycle	24	5.39	3.21	1.72E-06	0.002
	GO:0000279~M-phase	22	4.94	3.31	3.23E-06	0.005
	GO:0022403~cell cycle phase	24	5.39	2.87	1.11E-05	0.019
	GO:0022402~cell cycle process	28	6.29	2.45	2.87E-05	0.049
	GO:0051301~cell division	18	4.04	3.02	1.02E-04	0.175
VP1 in LoVo	GO:0006955~immune response	59	9.67	2.82	1.16E-12	0.000
	GO:0002376~immune system process	72	11.80	2.38	8.37E-12	0.000
	GO:0050896~response to stimulus	149	24.43	1.40	2.42E-06	0.004
VP2 in LoVo	No significant enrichment	-	-	-	-	-
NS1 in LoVo	GO:0048285~organelle fission	22	4.35	4.36	3.38E-08	0.000
	GO:0007067~mitosis	21	4.15	4.33	8.32E-08	0.000
	GO:0000280~nuclear division	21	4.15	4.33	8.32E-08	0.000
	GO:0000087~M-phase of mitotic cell cycle	21	4.15	4.26	1.12E-07	0.000
	GO:0000279~M-phase	25	4.94	3.45	2.75E-07	0.000
	GO:0000278~mitotic cell cycle	25	4.94	3.07	2.26E-06	0.004
	GO:0022403~cell cycle phase	26	5.14	2.85	4.98E-06	0.009
	GO:0022402~cell cycle process	30	5.93	2.41	1.99E-05	0.034
	GO:0051301~cell division	18	3.56	2.77	2.89E-04	0.492
Downregulation						
VP1 in HCEC	GO:0006520~cellular amino acid metabolic process	16	4.43	4.78	1.28E-06	0.002
	GO:0048037~cofactor binding	17	4.71	4.42	1.57E-06	0.002
	GO:0044106~cellular amine metabolic process	18	4.99	3.99	2.78E-06	0.004
	GO:0009308~amine metabolic process	20	5.54	3.38	7.38E-06	0.012
	GO:0019752~carboxylic acid metabolic process	24	6.65	2.92	7.81E-06	0.013
	GO:0043436~oxoacid metabolic process	24	6.65	2.92	7.81E-06	0.013
VP2 in HCEC	No significant enrichment	-	-	-	-	-
NS1 in HCEC	GO:0043167~ion binding	175	25.36	1.32	1.35E-05	0.020
	GO:0046872~metal ion binding	171	24.78	1.32	1.75E-05	0.026
	GO:0043169~cation binding	171	24.78	1.31	3.08E-05	0.046
VP1 in LoVo	GO:0006520~cellular amino acid metabolic process	14	4.27	4.58	1.17E-05	0.019
	GO:0009069~serine family amino acid metabolic process	6	1.83	17.06	2.19E-05	0.036
	GO:0046394~carboxylic acid biosynthetic process	11	3.35	5.24	4.67E-05	0.078
	GO:0016053~organic acid biosynthetic process	11	3.35	5.24	4.67E-05	0.077
	GO:0008652~cellular amino acid biosynthetic process	7	2.13	10.14	5.98E-05	0.099
VP2 in LoVo	No significant enrichment	-	-	-	-	-
NS1 in LoVo	No significant enrichment	-	-	-	-	-

HCEC, human colorectal epithelial cell; FDR, false discovery rate.

**Table V:** Significant pathways for differentially-expressed genes.

Group	Pathway name	Genes, n	Genes	Fold change	P-value	FDR
Upregulation						
VP1 in HCEC	Cell adhesion molecules	14	CD274, CD86, F11R, CDH1, ITGB8, ICAM1, HLA-A, HLA-B, HLA-C, HLA-E, HLA-F, HLA-G, HLA-DRB5, PTPRC, SDC4	2.4	0.005	5.9
	Antigen processing and presentation	12	B2M, CTSS, HSP70B, HSP70B′, HLA-A, HLA-B, HLA-C, HLA-E, HLA-F, HLA-G, HLA-DRB5, TAP1	2.7	0.010	1.1
	Cytokines and inflammatory response	7	CSF-2, CSF-3, IL1A, IL11, IL-6, IL-8, TNF	4.6	0.002	3.2
VP2 in HCEC	None					
NS1 in HCEC	Cell cycle	21	NDC80, SPC25, BUB1, CDC20, CENPM, CCNA2, CCND1, KIF20A, KIF23, MCM5, PTTG1, PTTG2, PSMB8, TUBA1A, TUBA4A, TUBB2C, TUBB, TUBB5, TUBBP2, TUBBP1, UBE2E1	7.0	0.001	1.3
	Pathways in cancer	9	BAX, APC, CCND1, FGF10, FGF17, LAMA4, RAC3, VEGFA, WNT10B	1.2	0.52	100
	Colorectal cancer	4	BAX, APC, RAC3, CCND1	2.0	0.32	99
	Focal adhesion	6	MYLPF, CAV1, CCND1, LAMA4, RAC3, VEGFA	1.5	0.34	99
VP1 in LoVo	Cell adhesion molecules	15	CD274, CD86, F11R, ITGB8, ICAM1, HLA-A, HLA-B, HLA-C, HLA-E, HLA-F, HLA-G, HLA-DRB5, SDC4, PVRL2, PVRL3	3.2	0.000	0.2
	Antigen processing and presentation	10	B2M, CTSS, HLA-A, HLA-B, HLA-C, HLA-E, HLA-F, HLA-G, HLA-DRB5, TAP1	3	0.009	10
	Cytokines and inflammatory response	4	CSF-3, IL1A, IL-8, TNF	2.9	0.015	84
VP2 in LoVo	None					
NS1 in LoVo	Not significant					
Downregulation						
VP1 in HCEC	Not significant					
VP2 in HCEC	None					
NS1 in HCEC	Not significant					
VP1 in LoVo	Not significant					
VP2 in LoVo	Not significant					
NS1 in LoVo	Cytoskeletal regulation by Rho GTPase	16	ASPM, ENAH, IGFN1, MYLK, MYH13, MYH6, PAK3, PAK2, RAC3, TTN, TUBB2A, TUBB3, TUBB, TUBB5, TUBBP2, TUBBP1,	2.2	0.013	13
	Wnt signaling pathway	22	ARID1A, EP300, INO80, SMARCB1, APC, ARRB2, DCHS1, FZD1, GNG3, MYH13, MYH6, NFATC3, PPP3CB, PCDH18, PCDH7, PCDHA5, PCDHGA5, PCDHGB7, SVEP1, TTBK1, TCF7L2, MTCL1	1.2	0.25	96
	Pathways in cancer	12	BAX, EP300, BMP4, FZD1, IGF1, PPARG, PIAS4, RAC3, RUNX1T1, TCEB2, TCF7L2, FOS	0.78	0.91	100
	Colorectal cancer	6	BAX, APC, RAC3, FZD1, TCF7L2, FOS	1.4	0.42	100

P-value, enrichment of differentially-expressed genes; FDR, false discovery rate; HCEC, human colorectal epithelial cell.

**Table VI:** Expression of 12 differentially-expressed genes detected using microarray analysis compared with qPCR analysis.

Gene symbol	Groups	qPCR	Microarray
FOSB	VP1/HCEC	1.6	1.7
MYBPC2	VP1/HCEC	−3.5	−3.2
APCDD1	VP2/HCEC	3.1	2.9
NME4	VP2/HCEC	−10.6	−11.2
RAC3	NS1/HCEC	1.7	1.4
TP53I11	NS1/HCEC	−4.0	−3.4
CRCX7	VP1/LoVo	1.5	1.6
ABCC6P1	VP1/LoVo	−3.7	−3.8
LRP11	VP2/LoVo	2.9	3.0
KRTAP21-1	VP2/LoVo	−6.9	−6.8
TCF7L2	NS1/LoVo	3.2	3.4
FOS	NS1/LoVo	−1.6	−1.8

Data are presented as the log^2^ ratio. HCECs, human colorectal epithelial cells; qPCR, quantitative polymerase chain reaction.
